# Effect of bioactive proteins on gait kinematics and systemic inflammatory markers in mature horses

**DOI:** 10.1093/tas/txab017

**Published:** 2021-02-08

**Authors:** K K Fikes, J A Coverdale, J L Leatherwood, J M Campbell, T H Welsh, C J Hartz, M Goehring, A A Millican, A N Bradbery, T A Wickersham

**Affiliations:** 1 Department of Animal Science, Texas A&M University, College Station, TX 77843; 2 APC, LLC, Ankeny, IA 50021, USA; 3 Department of Animal and Range Sciences, Montana State University, Bozeman, MT 59717, USA

**Keywords:** bioactive proteins, equine, gait kinematics, inflammation

## Abstract

Twenty-seven mature Quarter horses were used in a randomized design to determine the effects of bioactive protein supplementation on gait kinematics and systemic inflammatory markers in a 34-d trial. Treatments consisted of oral doses of 230 g/d of pelleted supplements containing 0 g (CON; *n* = 9), 40 g of bioactive protein (40BP; *n* = 9; LIFELINE, APC, LLC, Ankeny, IA), and 80 g of bioactive protein (80BP; *n* = 9) daily. Horses were fed a commercial concentrate at 0.5% BW (as-fed) and received *ad libitum* coastal bermudagrass (*Cynodon dactylon*) hay daily. On day 33, horses consistent in exercise (CON, *n* = 6; 40BP, *n* = 8; 80BP, *n* = 7) participated in a trailering and riding challenge. Kinematic gait analysis was performed on day 0 for use as a covariate, and on day 14, 28, and 34 to allow for the determination of potential time and dosage effects. Video footage was collected and analyzed using gait analysis software (EquineTec, Monroe, GA) for the determination of stride length (SL) and range of motion (ROM). Blood was collected via jugular venipuncture on days 0, 14, 28, and 34 for determination of systemic expression of tumor necrosis factor (TNF)-α and IL-1β. Data were analyzed using PROC MIXED of SAS. A trend towards treatment × time interaction was observed in ROM of the knee at the walk (*P* = 0.10), due to the increasing ROM for 40BP and 80BP as time increased and decreasing ROM for CON. A treatment × time interaction was observed (*P* < 0.01) for hock ROM at a walk resulting from CON and 80BP decreasing from day 14 to 28 with 40BP increasing, while from day 28 to 34 ROM at a walk decreased for 40BP and increased for 80BP. The main effect of treatment on hock ROM at the walk was quadratic (*P* < 0.01) and characterized by higher ROM values for 40BP compared to CON or 80BP. Dietary treatment lengthened (*P* = 0.04) SL of the hind limb at the walk for 40BP and 80BP compared to CON on both days 14 and 28. A significant treatment × time interaction was observed in the expression of IL-1β (*P* < 0.01) and can be explained by lower concentrations of IL-1β for 80BP on day 34 compared to the other treatments, with 40BP being intermediate and CON being the highest. Increased articular ROM with decreased expression of IL-1β may indicate potential anti-inflammatory effects of 80 g/d of bioactive proteins.

## INTRODUCTION

The performance potential of equine athletes is dependent upon the ability of articular joints to maximize the range of motion to create long, fluid strides. Data gathered through gait kinematic software are closely associated with local and systemic inflammatory responses which when elevated may the limit range of motion, stride length, and overall comfort of movement for the performance horse. Furthermore, excessive inflammation results in long-term damage to the joint and subsequent osteoarthritis, which is the greatest single economic loss to the equine industry (Frisbie et al., 2006). Therefore, developing nutritional strategies to mitigate the progression of joint inflammation and associated degenerative joint disease may reduce the need for oral or intra-articular pharmaceuticals, which act primarily to alleviate symptoms of pain associated with the disease.

A potential alternative is the use of dietary bioactive proteins (**BP**) derived from bovine spray-dried plasma proteins (**SDP**) to reduce the inflammatory effects associated with excess joint load and trauma. These proteins are a diverse mixture of functional components with biological activity independent of their nutritional value, including immunoglobulins, growth factors, biologically active peptides, and other factors with biological activity within the intestine (Campbell et al., 2010). Spray-dried plasma was found to be more successful than antibiotics in reducing the expression of pro-inflammatory cytokines in pigs challenged with *Escherichia coli* K88 (Bosi et al., 2004). Beski et al. (2016) observed that broiler chickens receiving SDP supplemented in starter diets had lower concentrations of immunoglobulins in serum, indicating that stimulation of the immune system was reduced, and more resources could be allocated to maintenance and development. Furthermore, in weaned rats challenged with *Staphylococcus aureus* superantigen B, dietary supplementation with SDP reduced immune activation of Peyer’s patches and mesenteric lymph nodes (Perez-Bosque et al., 2004).

More recent studies performed in horses suggest that BP improves gait kinematics in mature horses, specifically via improved mean stride length (**SL**) in the forelimb and hindlimb as levels of BP increase (Coverdale and Campbell, 2014). Systemic levels of key inflammatory biomarkers in serum provide an indication of total metabolic activity originating from all joints in the body (Sumer et al., 2006). To date, limited work has been performed to determine the effects of dietary BP supplementation in horses on the relationship between blood chemistry parameters, serum inflammatory biomarkers, and performance gait kinematics. Therefore, the objective of this study was to determine the effect of dietary BP supplementation on gait kinematics and systemic inflammatory markers in horses over time and following an extensive transport and exercise challenge. We hypothesized that dietary BP will reduce basal inflammation and the inflammatory response to a transport and exercise challenge resulting in improved gait kinematics and overall performance potential.

## MATERIALS AND METHODS

### Horses and Dietary Treatments

All procedures and handling of horses were approved by the Institutional Animal Care and Use Committee (AUP# 2015-0380). Twenty-seven mature horses (*n* = 25 geldings, *n* = 2 mares) from an established herd at Parsons Mounted Cavalry (Texas A&M University, College Station, TX) were utilized in a randomized design for a 34-d trial. Horses were housed in a dry-lot track (9 m × 1600 m) for voluntary exercise. All horses were allowed *ad libitum* access to coastal Bermudagrass hay (*Cynodon dactylon*) obtained from a single cutting and water. Horses were fed a commercially available concentrate (14% CP pellet; Cargill, Elk River, MN) twice daily in 3 m × 3 m individual box stalls every 12 h. Horses underwent moderate exercise for the duration of the study in accordance with Parsons Mounted Cavalry protocol, consisting of basic horsemanship maneuvers at the walk, trot, and canter 5 d/wk for approximately 60 min each day.

before the start of the experimental period, horses were stratified by body weight (**BW**; 560 ± 52 kg), body condition score (**BCS**; 5.21 ± 0.42), and age (13 ± 3 yr) and randomly assigned to one of three dietary treatment groups that included a supplement containing either soybean meal or two levels of spray-dried bioactive proteins (LIFELINE Equine Elite, BioThrive, APC, LLC, Ankeny, IA). Dietary pelleted supplements were top-dressed (230 g/d) on the commercially available concentrate feed (Table 1) and consisted of a control soy protein-based supplement without BioThrive (**CON**; *n* = 9), 40 g BioThrive (**40BP**; *n* = 9), or 80 g BioThrive (**80BP**; *n* = 9) with BioThrive replacing soy protein in the respective supplement. Investigators remained blinded to treatments until completion of data analysis, and treatments were coded alphabetically and pre-weighed to 230 g. Dietary treatments began on day 1, and no significant refusals were recorded after day 3. Horses were allowed 1 h to consume dietary treatments before orts were collected.

**Table 1.** Nutrient analysis of dietary treatments including commercial concentrate and top-dressed supplement

	Concentrate^*a*^	CON	40BP^*b*^	80BP^*b*^
Dry Matter, %	90.0	91.9	93.0	91.8
	DM Basis, %			
CP	17.9	32.6	34.4	38.8
NDF	28.7	32.9	32.4	32.7
ADF	16.6	23.0	22.4	21.4
Ca	1.17	1.58	1.68	1.74
P	0.99	1.09	0.99	0.86

^a^14% CP pelleted feed (Cargill, Elk River, MN).

^b^LIFELINE Equine Elite (APC, LLC, Ankeny, IA).

On day 33, horses that remained consistent in exercise (CON: *n* = 6; 40BP: *n* = 8; 80BP: *n* = 7) underwent an exercise stressor via participation in the Battle of Flowers Parade in San Antonio, TX. Horses have hauled 290 km in a stock-type trailer with approximately 1 h rest before walking a 6.5 km parade on concrete followed by a 290 km return in the same stock-type trailer. Previous research has indicated limited stress to joints on a sandy surface compared to asphalt and has also indicated changes to gait kinematics on an asphalt surface when compared to sand (Chateau et al., 2010; Chateau et al., 2013). Further, transport has been shown to induce inflammation in both cattle and horses (Wessely-Szponder et al., 2015; Van Engen and Coetzee, 2018).

### Sample Collection

Weekly, BW and BCS were determined using a calibrated platform scale and three independent evaluators, respectively (Henneke et al., 1983). Plasma and serum samples were collected every 7 d from day 0 to 28, and 24 h post-parade on day 34. Approximately 50 mL of whole blood was collected via jugular venipuncture. Samples intended for plasma analysis were collected into 10 mL evacuated tubes containing 0.1 mL 15% buffering solution and 15 mg K_3_ EDTA (BD Vacutainer, Franklin Lakes, NJ) and immediately placed in ice. Samples intended for serum analysis were collected into 10 mL evacuated non-additive tubes (BD Vacutainer, Franklin Lakes, NJ) and allowed to clot for 30 min before processing to allow blood to clot. All samples were centrifuged at 2,000 × *g* at 4°C for 20 min. The supernatant was transferred to pour off tubes and placed into a freezer at −20°C for subsequent analysis.

### Gait Kinematics

Gait analysis was performed every two weeks on day 0, 14, and 28, and 24 h post-parade on day 34. Horses were assigned a single handler for each gait analysis throughout the study. Horses performed three passes at both the walk and trot over a 10 m path on solid dirt footing. Reflective adhesive markers were placed on major joints on the right front and right hind limb to allow for visibility and calibration for later analysis. Front limb markers included the greater tubercle of the humerus, lateral humeral epicondle, ulnar carpal bones, lateral metacarpal epicondyle, middle phalanx-proximal phalanx junction, and proximal phalanx-distal phalanx junction. Hind limb markers included distal phalanx-middle phalanx junction, middle phalanx-proximal phalanx junction, proximal phalanx- third metacarpal junction, tarsal bones, and lateral femoral epicondle. Video footage was collected and recorded using EquineTec software (EquineTec, Monroe, GA) installed on an HP Pavilion m6 laptop with a 2.3 GHz microprocessor and AMD Rodeon HD 7660G video graphics (Hewlett-Packard, Palo Alto, CA).

### Sample Analysis

Serum collected was analyzed for tumor necrosis factor α (**TNF-α**) and interleukin-1β (**IL-1β**) using commercially available ELISA kits (R&D Systems, Inc., Minneapolis, MN) developed for use in the horse. Assays were run according to the kit insert. The inter- and intra-assay CV was ≤ 10% for both TNF-α and IL-1β. Plasma samples were used for blood chemistry profiles where abnormal hematology may indicate internal disease independent of joint disease. Samples were analyzed by the Texas Veterinary Medical Diagnostic Lab at Texas A&M University (College Station, TX) for total serum protein (**TSP**), albumin, phosphorus (**P**), glucose, blood urea nitrogen (**BUN**), creatinine, creatine kinase (**CK**), total bilirubin (**TB**), bilirubin direct (**BD**), gamma-glutamyl transferase (**GGT**), albumin:globulin (**A/G**) ratio, and globulins. Horses with values outside of normal ranges were excluded from cytokine analysis to ensure that expression of systemic inflammatory markers was reflective of joint disease and no other inflammatory processes. One horse from each treatment group, CON, 40BP, and 80BP were removed from cytokine analysis due to abnormal blood chemistry parameters not explained by joint disease.

### Statistical Analysis

All data were analyzed as a randomized design using the PROC MIXED procedure of statistical analysis software (SAS) (SAS Inst., Inc., Cary, NC) with effects for treatment, day, and treatment × day interaction with linear and quadratic contrasts. The model uses RANDOM and REPEATED statements to account for variability between animals. Day 0 was used as a covariate for gait kinematics. Effects were considered significant if *P* ≤ 0.05, with a trend towards significance if *P* ≤ 0.10.

## RESULTS

### Physical Measurements

**Figure 1.**
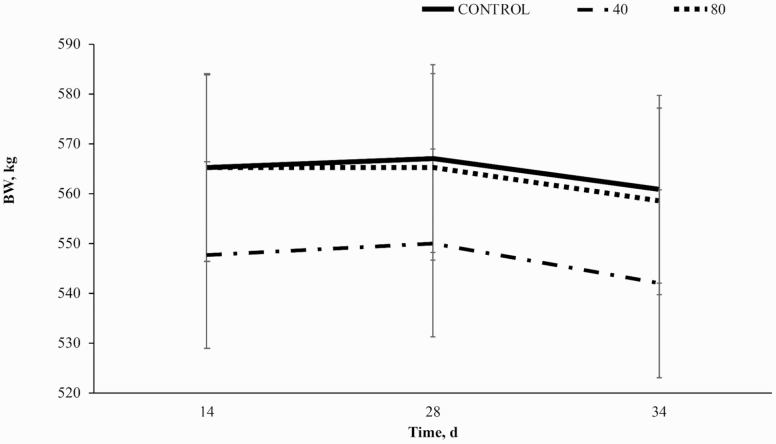
Bodyweight (kg) over time (d) in horses receiving a pelleted concentrate with control, 40BP, or 80BP g/d of spray-dried bioactive proteins (LIFELINE, APC, LLC, Ankeny IA). Main effects included treatment, time, and treatment × time.

**Figure 2.**
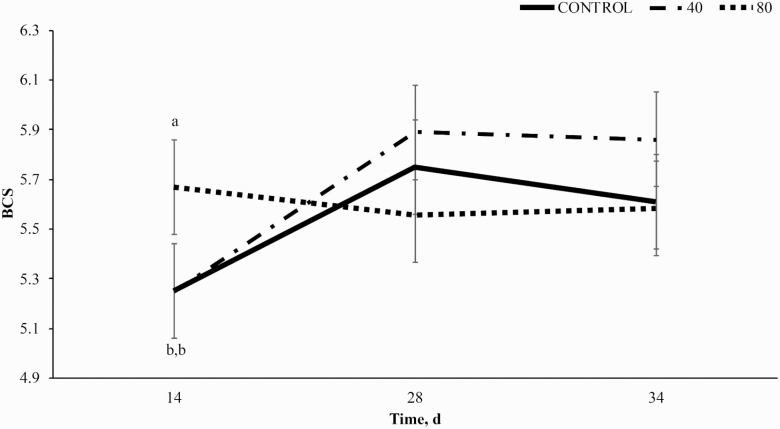
Body condition score (BCS) over time (d) in horses receiving a pelleted concentrate with either control, 40BP, or 80BP g/d of spray-dried bioactive proteins (LIFELINE, APC, LLC, Ankeny IA). Main effects included treatment, time, and treatment × time. ^a,b,c^ Within d.

There was no effect of BP supplementation on BW (*P* = 0.76; Fig. 1); however, there was a treatment × time interaction for BCS (*P* < 0.01; Fig. 2) with 80BP having greater BCS on day 14 compared to both 40BP and CON. Horses receiving 80BP maintained BCS through day 28, while CON and 40BP increased BCS such that there was no significant difference between dietary treatments on d 28. All horses, regardless of dietary treatment, gained BW (*P* < 0.01) until day 28 and lost BW to day 34 while BCS remained constant.

### Gait Kinematic Parameters

#### Stride length:

**Figure 3.**
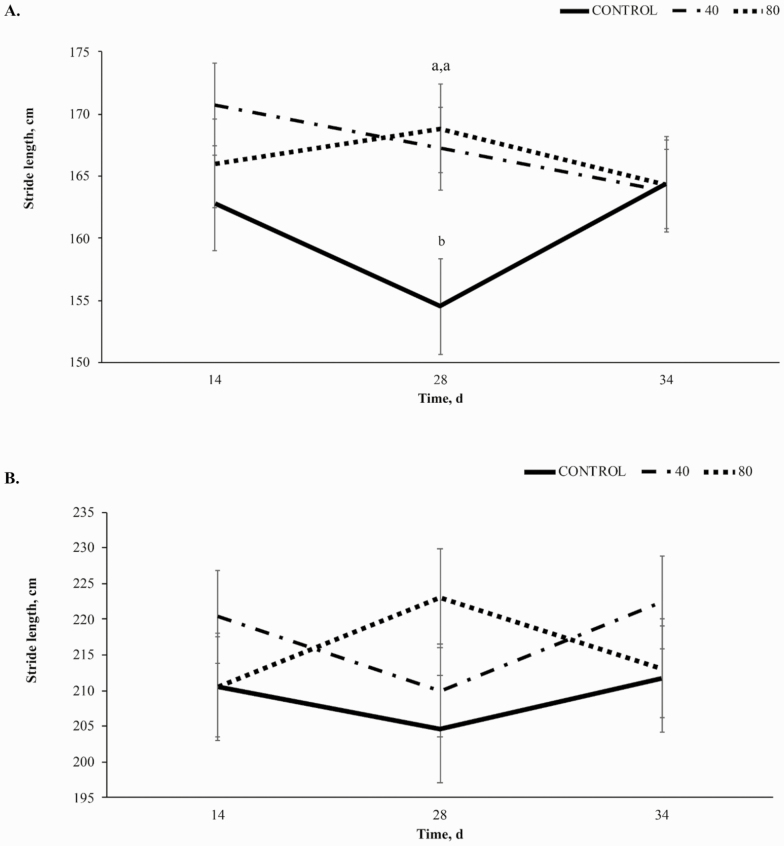
Stride length (cm) of the forelimb at the walk (A) and trot (B) over time (d) in horses receiving a pelleted concentrate with either control, 40BP, or 80BP g/d of spray-dried bioactive proteins (LIFELINE, APC, LLC, Ankeny IA). Main effects included treatment, time, and treatment × time. ^a,b,c^Within d, superscripts denote a difference between dietary treatments (*P* < 0.05).

A trend towards a treatment × time interaction (*P* = 0.10, Fig. 3A) was observed in stride length (**SL**) of the forelimb at the walk, largely driven by an increase in SL in horses receiving 80BP while horses receiving CON and 40BP decreased from d 14 to d 28. A trend towards a linear effect (*P* = 0.09) was also demonstrated with CON SL at 155 ± 3.83 cm on d 28, 40BP at 168 ± 5.0 cm, and 80BP at 168.9 ± 3.56 cm. No significant effects were observed for SL of the forelimb at the trot (*P* = 0.21, Fig. 3B).

**Figure 4.**
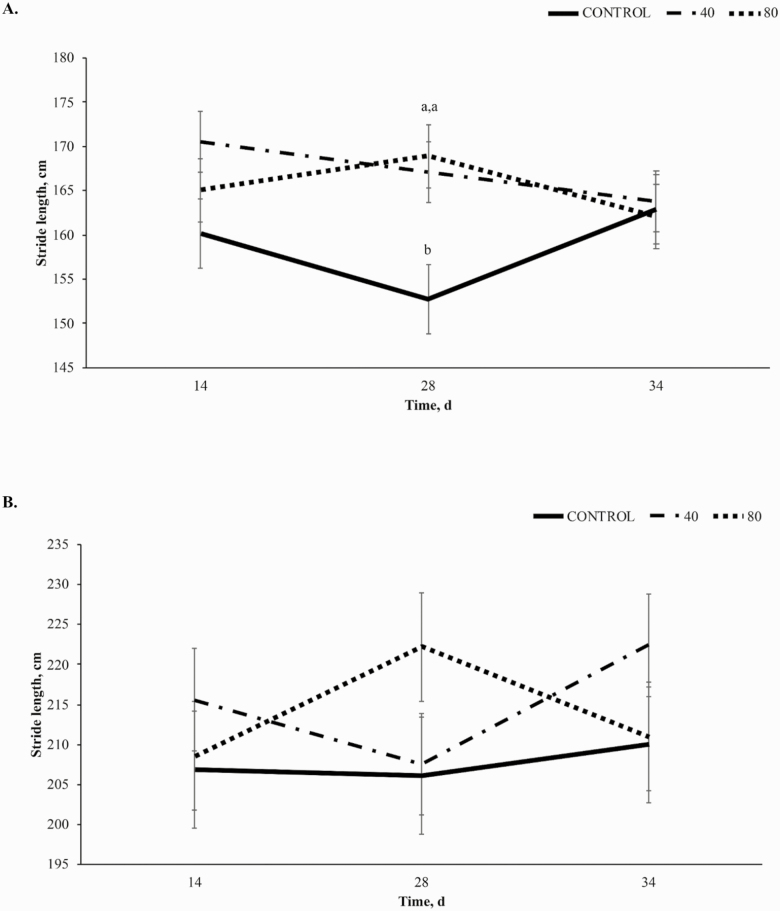
Stride length (cm) of the hind limb at the walk (A) and trot (B) over time (d) in horses receiving a pelleted concentrate with either control, 40BP or 80BP g/d of spray-dried bioactive proteins (LIFELINE, APC, LLC, Ankeny IA). Main effects included treatment, time, and treatment × time. ^a,b,c^Within d, superscripts denote a difference in dietary treatment (*P* < 0.05).

At the walk, a trend towards a treatment × time interaction (*P* = 0.07; Fig. 4A) was observed for hindlimb SL, resulting from decreases in SL from day 14 to 28 in CON and 40BP while 80BP increased. From day 28 to 34, 40BP and 80BP decreased while CON increased. There was a linear increase in SL of the hindlimb at the walk-in treated horses compared to CON (*P* = 0.05). Treatment did not affect SL of the hindlimb at the trot (*P* = 0.17; Fig. 4B).

#### Range of motion.

**Figure 5.**
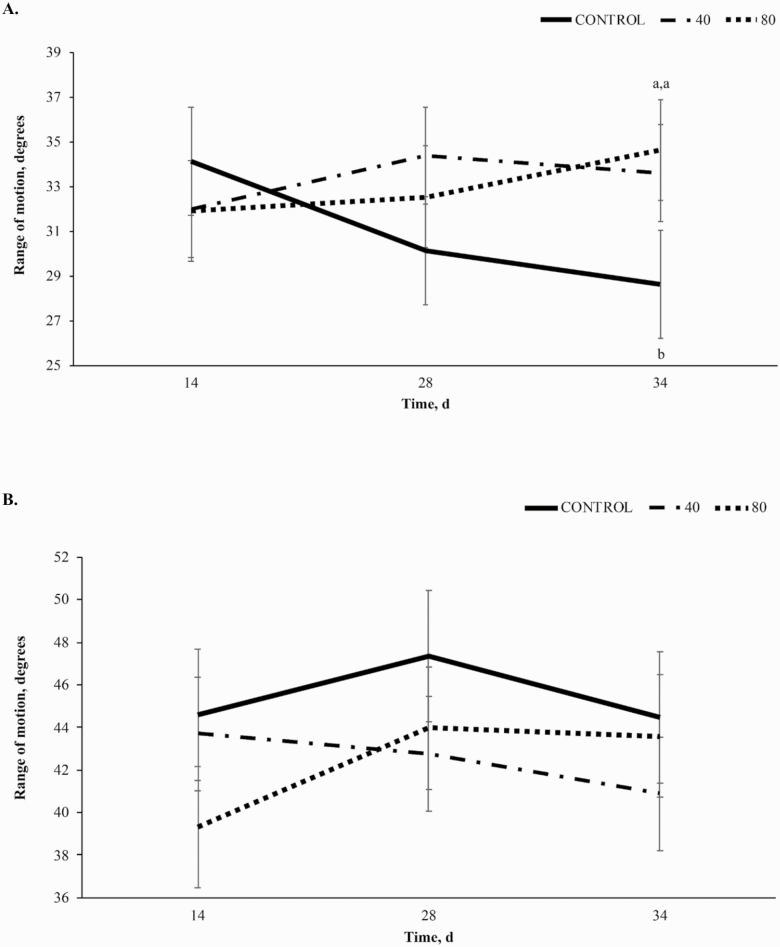
Range of motion (degrees) of the knee at the walk (A) and trot (B) over time (d) in horses receiving a pelleted concentrate with either control, 40BP, or 80BP g/d of spray-dried bioactive proteins (LIFELINE, APC, LLC, Ankeny IA). Main effects included treatment, time, and treatment × time. ^a,b,c^Within d, superscripts denote a difference in dietary treatment (*P* < 0.05).

Range of motion (**ROM**) in the knee at the walk exhibited a trend towards a treatment × time interaction (*P* = 0.10, Fig. 5A), due to the increased range of motion for treated horses compared to CON on day 34. Horses in the CON dietary treatment decreased ROM from day 14 to 34, while treated horses increased. Horses receiving 80BP increased ROM throughout the study, with ROM reaching 32.5 ± 2.27° on day 28 and 34.6 ± 2.27° on day 34. The 40BP dietary treatment group increased ROM to 34.4 ± 2.16° on day 28 and decreased to 33.6 ± 2.16° on day 34. Supplementation of BP did not influence knee ROM at the walk or trot (*P* = 0.56, *P* = 0.38, respectively). No significant effects were observed for knee ROM at the trot (*P* = 0.64; Fig. 5B).

**Figure 6.**
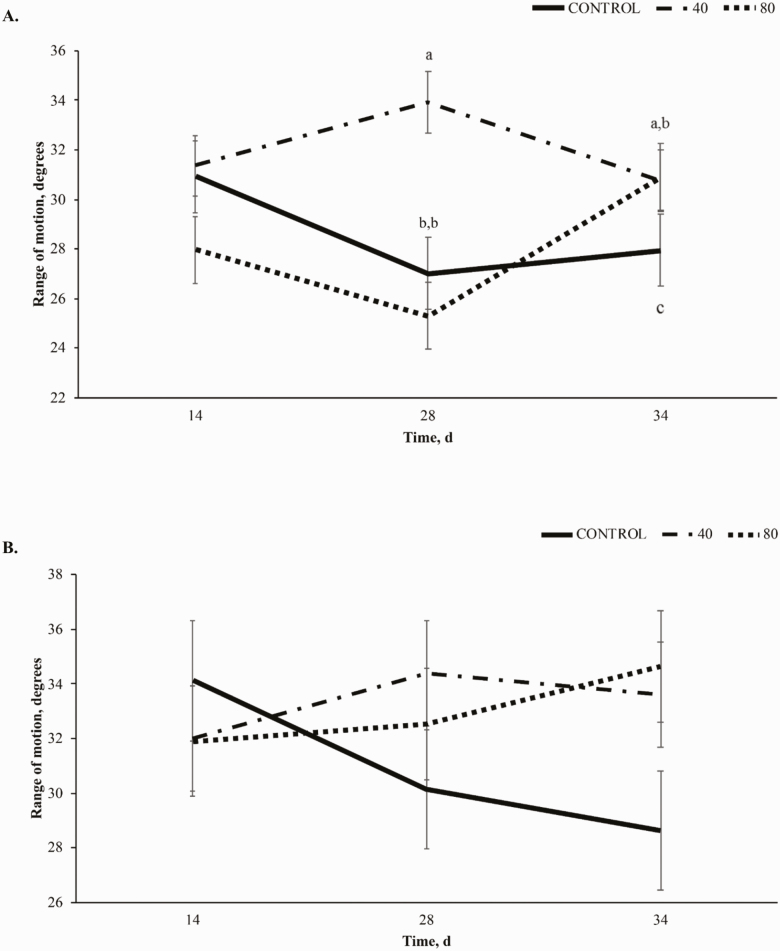
Range of motion (degrees) of the hock at the walk (A) and trot (B) over time (d) in horses receiving a pelleted concentrate with either control, 40BP, or 80BP g/d of spray-dried bioactive proteins (LIFELINE, APC, LLC, Ankeny IA). Main effects included treatment, time, and treatment × time. ^a,b,c^Within d, superscripts denote differences in dietary treatment (*P* < 0.05).

A significant treatment × time interaction (*P* < 0.01, Fig. 6A) was observed for hock ROM at the walk described by an increase in ROM in 80BP horses from d 28 to d 34, a decrease from d 28 to 34 in 40BP horses, and relatively constant values in CON horses. Dietary supplementation of BP affected hock range of motion at the walk quadratically (*P* < 0.01) demonstrated by 40BP having the highest ROM at 33.2 ± 1.24°, followed by CON at 28.6 ± 1.45°, and 80BP having the lowest ROM at 28.0 ± 1.36°. Hock ROM at the trot was influenced by time (*P* = 0.05 Fig. 6B), but not treatment (*P* = 0.39) with all horses decreasing ROM over time.

#### Blood chemistry parameters.

**Figure 7.**
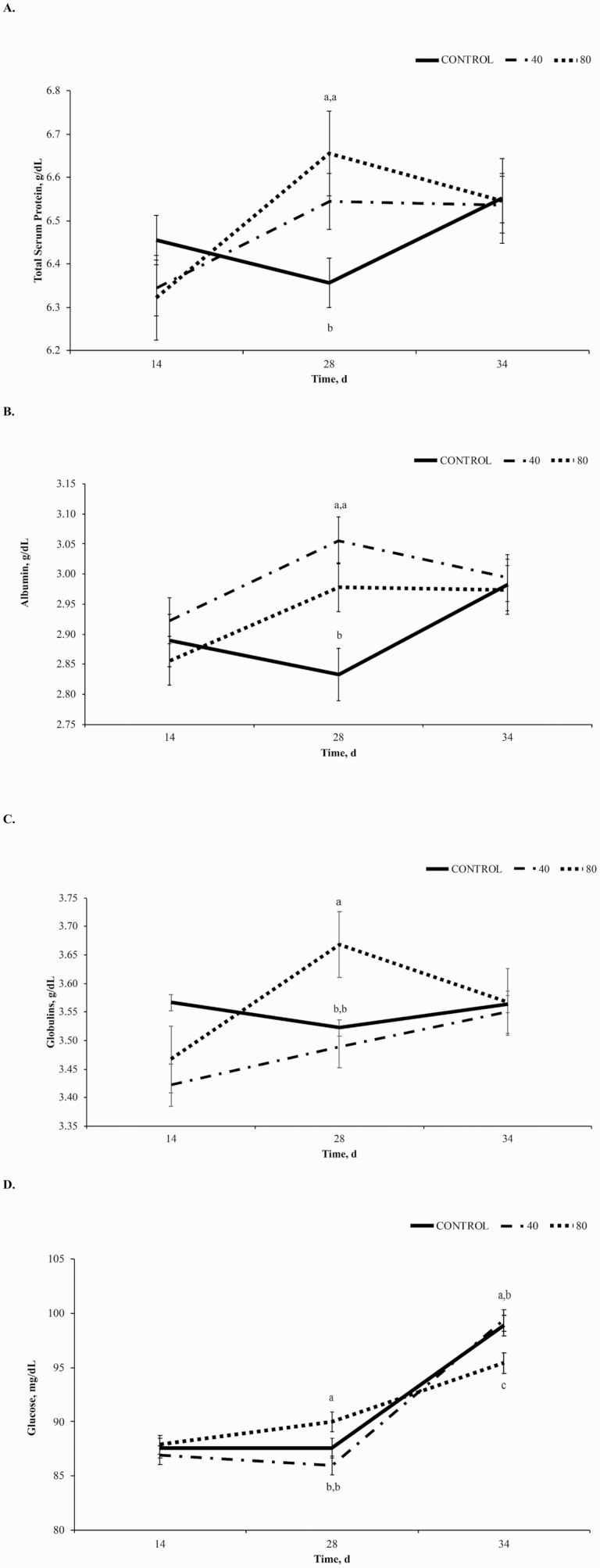
The effect of dietary supplementation of control, 40BP or 80BP g/d of spray-dried bioactive proteins (LIFELINE, APC, LLC, Ankeny IA) on total serum protein (A; g/dL), albumin (B; g/dL), globulins (C; g/dL), and blood glucose (D; mg/dL). Main effects included treatment, time, and treatment × time. ^a,b,c^Within d, superscripts denote a difference in dietary treatment (*P* < 0.05).

Dietary supplementation of BP did not influence blood chemistry parameters (*P* > 0.10, Table 2); however, treatment × time interactions were observed for TSP, albumin, globulins, and glucose (*P* < 0.09). The interaction for TSP (*P* = 0.04; Fig. 7A) resulted from a decrease in TSP in CON with a corresponding increase in BP supplemented horses to day 28. The interaction of treatment × time for albumin (*P* < 0.01; Fig. 7B) is described by a decrease in CON to d 28 while albumin increased in treated horses. Globulins tended to express a treatment × time interaction (*P* = 0.09; Fig. 7C) where 40BP and 80BP horses increased to day 28 and CON horses decreased. Finally, a treatment × time interaction for glucose (*P* = 0.02; Fig. 7D) is described by lower plasma blood glucose on day 34 in 80BP horses compared to CON or 40BP.

**Table 2.** Least square treatment means of blood chemistry including total serum protein (TSP), albumin, phosphorus (P), glucose, blood urea nitrogen (BUN), creatinine, total bilirubin (TB), bilirubin direct (BD), creatine kinase (CK), serum glutamine oxaloacetate transaminase (SGOT), globulins, albumin:globulin ratio (A:G), and gamma-glutamyl transferase (GGT) in plasma of horses receiving a pelleted concentrate with either 0 g/d, 40 g/d, or 80 g/d of bioactive proteins

					*P*-values		
Parameter	0 g/d	40 g/d	80 g/d	SEM	Treatment	Time	Treatment x Time
TSP, g/dL	6.45	6.48	6.51	0.13	0.96	0.01	0.04
Albumin, g/dL	2.9	2.99	2.94	0.04	0.33	<0.01	<0.01
P, mg/dL	2.9	2.88	2.93	0.08	0.89	<0.01	0.99
Glucose, mg/dL	91.33	90.74	91.1	1.29	0.95	<0.01	0.02
BUN, mg/dL	15.52	16.44	15.86	0.62	0.57	<0.01	0.17
Creatinine, mg/dL	1.37	1.43	1.41	0.05	0.72	<0.01	0.17
TB, mg/dL	0.7	0.76	0.61	0.05	0.15	<0.01	0.09
BD, mg/dL	0.27	0.28	0.27	0.01	0.72	<0.01	0.56
CK, U/L	302.73	307.67	306.29	24.35	0.99	0.27	0.48
SGOT, U/L	274.83	255.61	278.9	12.76	0.4	0.63	0.38
Globulins, g/dL	3.55	3.49	3.57	0.15	0.92	0.09	0.09
A:G	0.81	0.89	0.84	0.04	0.42	0.89	0.11
GGT, U/L	14.05	11.51	11.89	1.35	0.37	0.02	0.15

#### *Serum inflammatory markers*.

**Figure 8.**
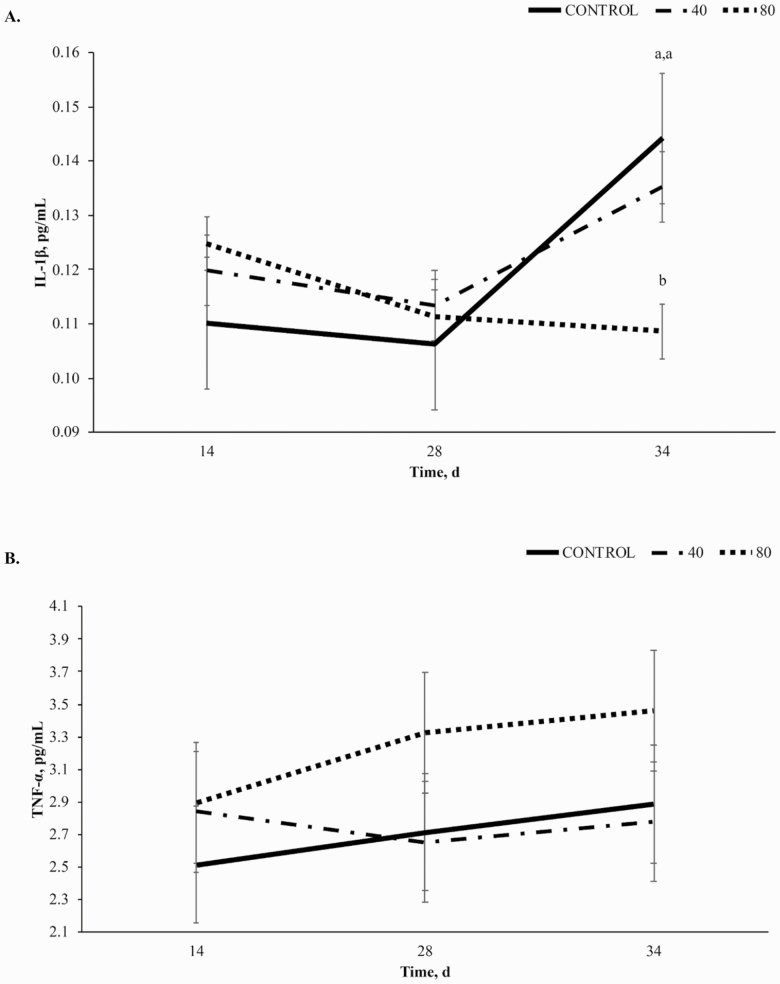
Serum concentrations of interleukin-1β (IL-1β; A; pg/mL) and tumor necrosis factor α (TNFα; B; pg/mL) over time (d) in horses receiving a pelleted concentrate with either control, 40BP, or 80BP g/d of spray-dried bioactive proteins (LIFELINE, APC, LLC, Ankeny IA). Main effects included treatment, time, and treatment × time. ^a,b,c^Within d, superscripts denote differences between dietary treatments for serum IL-1β (*P* < 0.05).

A treatment × time interaction was observed for IL-1β (*P* < 0.01; Fig. 8A) is described by CON and 40BP horses increasing to d 34 while 80BP horses decreased expression of IL-1β. Dietary treatment of BP did not affect serum levels of TNF-α (*P* = 0.51; Fig. 8B); however, TNFα increased over time regardless of dietary treatment (*P* = 0.05), with all horses increasing from d 28 to d 34. Time also influenced IL-1β (*P* < 0.01) as all treatments decreased to day 28.

## DISCUSSION

This study examined the effect of dietary BP supplementation on gait kinematics and systemic inflammatory markers in horses over time and following an extensive transport and aerobic exercise challenge. Previous research in horse suggests that dietary BP improves stride length which indicates increased levels of comfort (Coverdale and Campbell, 2014). This study is the first in horses to measure systemic markers of inflammation with gait kinematics in response to dietary BP supplementation and exercise.

Increased BW over time to day 28 may be explained by the change from group feeding to individual feeding at the initiation of the study to ensure each horse was fed to meet NRC requirements for mature horses undergoing light exercise (NRC, 2007). Minor loss in BW between day 28 and 34 is likely due to trailering and participation in the parade challenge on day 34 of the study. Decreased BW may be related to body water loss via sweat and subsequent dehydration as horses were weighed 24 h following transport (Friend, 2000). A study performed by Friend (2000) observed a similar initial loss in BW during transport, which was sustained but not increased for 24 h in horses with access to water.

Improvements in SL in the forelimb to day 28 at the walk were similar to those observed by Coverdale and Campbell (2014, 2015). This study included a challenge component that incorporated trailering and riding on day 33, leading to the overall trends being altered from previous studies that did not include a trailering and exercise challenge. Forelimb and hindlimb SL at the walk had a trend toward significance in this study compared to forelimb and hindlimb SL at the trot. This may be explained by the varying biomechanics of movement for the different gaits. Because the walk lacks a suspension phase (Clayton, 1989), training and coordination of the individual horse play a smaller role. This would allow any potential benefits of BP supplementation to become more evident and less reliant on individual athletic ability.

Bioactive proteins generally modulate the immune response to physiological stress in a challenging environment (Bosi et al., 2004; Perez-Bosque et al., 2016). However, SL for both limbs at the walk decreased on day 34. The inconsistent response may be explained by other factors influencing SL after the challenge, such as muscle and periarticular tissue soreness and behavior factors during the challenge (Rawson et al., 2001; Lee et al., 2002; Braun and Dutto, 2003). Additionally, during gait kinematic analysis, horses were recorded at the walk before moving into the trot, potentially acting as a warm-up to exercise, allowing the horse to become more fluid in the movements at the trot. While little work has been done investigating the effects of a walking warmup on equine gait kinematics, in humans, walking improves gait biomechanics thus it is likely that similar effects are present in this study (Schieber et al., 2019). Furthermore, in the instance of muscle soreness following transport and exercise stress, the active walking warmup may have reduced any incidence of delayed onset muscle soreness (Andersen et al., 2013).

The interaction observed for knee ROM at the walk but not at the trot may also be attributed to differing gait biomechanics as well as individual athletic ability. Previous studies did not observe trends or significance in knee ROM at either gait (Coverdale and Campbell, 2014, 2015). The response observed in the present study for this parameter of gait kinematic analysis to the challenge on day 33 indicates that treated horses improved in performance compared to CON. Articular ROM relates more closely to periarticular joint comfort than SL, suggesting that bioactive protein supplementation was more effective in improving articular ROM than SL following a challenge (Bischoff et al., 2003; Ahvazi et al., 2014).

Currently, there is no literature on gait kinematic analysis of hock ROM as it relates to SDP supplementation. It is unknown why horses receiving 40 g/d BP showed improved hock ROM at the walk to day 28 compared to horses receiving 80 g/d BP. However, hock ROM at the walk responded to the day 33 challenge similarly to knee ROM at the walk with horses receiving 80 g/d BP having improved articular ROM in response to the challenge, further suggesting that BP are more effective at improving articular ROM than SL after an exercise challenge.

Total serum protein (TSP) is a combined measure of all proteins in the blood, including albumin and globulins. Albumin is a blood protein produced by the liver and is integral in the maintenance of osmotic pressure (Miglio et al., 2019). Globulins include antibodies and other proteins involved in blood clotting and inflammation comprising many key components of the immune response (Miglio et al., 2019). Because SDP contains functional factors such as immunoglobulins, growth factors, and active peptides (Campbell et al. 2010b), it is possible that levels of total serum protein, albumin, and globulins would increase in treated horses either by differences in dietary crude protein (CP; Leveille and Sauberlich, 1961) or bioactive protein stimulation of the common mucosal system to result in increased globulin concentrations (Walther and Sieber, 2011).

Decreasing or sustained levels of total serum protein, albumin, and globulins to day 34 in treated horses compared to CON, whose levels increased from day 28 to 34 following the day 33 challenge, may indicate modulation of the immune response following physiological stress of transport and exercise. Furthermore, previous studies have observed modulatory effects of SDP on plasma glucose resulting from alterations in SGLT1 expression in a challenging environment and an increase in glucose uptake by insulin-sensitive tissues (Moreto and Perez-Bosque, 2009; Oseguera-Toledo et al., 2014). Our study shows comparable results following the day 33 challenge where horses receiving 80BP had lower plasma blood glucose than CON or 40BP.

In other species, SDP has demonstrated the ability to regulate cytokine expression (Bosi et al., 2004; Perez-Bosque et al., 2016). Following an acute lipopolysaccharide challenge in swine, SDP decreased mucosal expression of TNF-α (Bosi et al., 2004). However, in the current study, serum TNF-α levels were not influenced by dietary treatment. This is likely due to the use of a physical stressor rather than a pathogen-induced challenge and the use of a sustained physical challenge rather than an acute response. Pathogen recognition functions through acquired immunity, while acute physical stress functions primarily through the innate immune system. This change could explain the difference in response between the current study and the study performed by Bosi et al. (2004). Furthermore, rather than testing an acute response, sampling in the present study was designed to test the response to a sustained physical challenge and resulted in improved hock ROM suggesting anti-inflammatory effects.

Similar to the Perez-Bosque et al. (2016) mouse model, the current study demonstrates decreased levels of IL-1β in horses receiving 80BP following the day 33 challenge. Horses receiving 80BP had increasing values of TNF-α to day 28 and 34 with decreasing values of IL-1β. This suggests an improved ability to adapt to the stress-related immune response before IL-1β is expressed. Because IL-1β is the major cytokine associated with joint disease, this also suggests that dietary SDP’s observed improvement of articular ROM may be explained by the reduced expression of pro-inflammatory IL-1β.

An additional explanation for the modulation of IL-1β and not TNF-α on day 34 involves the timing of sample collection. Blood samples were harvested 24 h after the day 33 challenge. Because TNF-α levels are highest during acute inflammation, this amount of elapsed time from challenge to time of sampling in our study may have been insufficient for horses to begin resolution of any ongoing inflammatory response (McIlwraith et al., 2012). Levels of IL-1β fluctuate between acute and chronic inflammation, but remain present, allowing for any modulatory effects of dietary SDP to be observed 24 h after the challenge event.

In summary, the present study suggests that dietary BP supplementation improves SL of the hindlimb and ROM of the hock at the walk. Related to forelimb kinematic movements, treatment by time interactions suggest a trend towards improved forelimb SL and ROM. Blood chemistry profiles expressed differences in treatment response over time for TSP, albumin, globulins, and glucose consistent with previously published literature and supporting the efficacy of dietary BP. These data are further supported by immunomodulatory effects via IL-1β over time and in response to a transport and exercise challenge. This study is among the first to describe the systemic influence of dietary BP supplementation on inflammation and its effects on equine gait kinematics related to performance potential.

## LIST OF ABBREVIATIONS

SDP: spray-dried plasma protein
BP: a proprietary blend of bovine plasma fractions including serum-based bioactive proteins
BW: body weight
BCS: body condition score
CON: control dietary treatment
40BP: 40 g/d spray-dried bioactive protein supplement
80BP: 80 g/d spray-dried bioactive protein supplement
TNFα: tumor necrosis factor α
IL-1β: interleukin-1β
TSP: total serum protein
P: phosphorus
BUN: blood urea nitrogen
CK: creatine kinase
TB: total bilirubin
BD: bilirubin direct
GTT: gamma-glutamyl transferase
A/G: albumin:globulin
SL: stride length
ROM: range of motion
